# Association between Temperature and Influenza Activity across Different Regions of China during 2010–2017

**DOI:** 10.3390/v15030594

**Published:** 2023-02-21

**Authors:** Dina Wang, Hao Lei, Dayan Wang, Yuelong Shu, Shenglan Xiao

**Affiliations:** 1 School of Public Health (Shenzhen), Sun Yat-sen University, Guangzhou 510275, China; 2 School of Public Health (Shenzhen), Shenzhen Campus of Sun Yat-sen University, Shenzhen 518107, China; 3 School of Public Health, Zhejiang University, Hangzhou 310058, China; 4 National Institute for Viral Disease Control and Prevention, Collaboration Innovation Center for Diagnosis and Treatment of Infectious Diseases, Chinese Center for Disease Control and Prevention, Beijing 102206, China; 5 Institute of Pathogen Biology of Chinese Academy of Medical Science (CAMS)/Peking Union Medical College (PUMC), Beijing 100730, China

**Keywords:** temperature, influenza A, influenza B, influenza-like illness

## Abstract

Influenza causes a significant disease burden as an acute respiratory infection. Evidence suggests that meteorological factors can influence the spread of influenza; however, the association between these factors and influenza activity remains controversial. In this study, we investigated the impact of temperature on influenza across different regions of China based on the meteorological data and influenza data from 554 sentinel hospitals in 30 provinces and municipalities in China from 2010 to 2017. A distributed lag nonlinear model (DLNM) was used to analyze the exposure lag response of daily mean temperatures to the risk of influenza-like illness (ILI), influenza A (Flu A), and influenza B (Flu B). We found that in northern China, low temperatures increased the risk of ILI, Flu A, and Flu B, while in central and southern China, both low and high temperatures increased the risk of ILI and Flu A, and only low temperatures increased the risk of Flu B. This study suggests that temperature is closely associated with the influenza activity in China. Temperature should be integrated into the current public health surveillance system for highly accurate influenza warnings and the timely implementation of disease prevention and control measures.

## 1. Introduction

Influenza is an acute respiratory infection caused by the influenza virus, which brings in a considerable disease burden upon economies and societies worldwide. Two subtypes of influenza viruses, Flu A and Flu B viruses, circulate and cause seasonal epidemics. As estimated by the World Health Organization (WHO), seasonal influenza epidemics cause 3–5 million severe cases and about 290,000–650,000 respiratory disease-related deaths worldwide annually [1]. The cumulative number of influenza infections in China reached 89.453 million during 2010–2020 [2]. Since 1952, influenza has been under international surveillance designated by the WHO to monitor influenza activity globally and develop prevention and control strategies.

Researchers have long studied how the climate affects the spread of influenza [3,4]. There is substantial evidence that meteorological and environmental conditions such as temperature and humidity can influence the dynamics of influenza virus transmission. Low temperatures favor the spread of influenza in temperate and subtropical regions [5]. In Hong Kong, rising temperatures may also increase the incidence rate of Flu A [6]. Several laboratory and animal studies have demonstrated an increased efficiency of influenza virus transmission in colder environments [7,8] and longer survival of the virus in vitro [9]. Additionally, some studies have pointed out that the epidemic characteristics of the two influenza virus subtypes, Flu A and Flu B, are different [10]. In mainland China and Hong Kong [11], Flu A outbreaks occurred in both winter and summer, while Flu B outbreaks occurred in winter. Although the biological mechanism of the seasonal difference between these two subtypes has not been elucidated, epidemiological results showed that the survival and transmission of the two subtypes varied under different environmental conditions [12]. Understanding the patterns of influenza virus transmission and outbreaks is key to preventing influenza; therefore, the drivers of influenza need to be explored in depth.

At present, most studies are limited to a specific region or province and national analysis reports are rare, so the representativeness of their conclusions is not good enough. China is a country that includes tropical and temperate zones, with a vast territory and diverse climate; therefore, there are large differences in temperature, precipitation, and other parameters in areas. The timing of seasonal influenza epidemics varies by latitude, with influenza occurring in winter in temperate regions and year-round in tropical regions [13]. Hence, from a public health perspective, assessing the impact of temperature on influenza activity at the national level and identifying its causes are of great importance for developing targeted influenza control and prevention measures. In this study, the lag relationship between daily mean temperature and the transmission and incidence of influenza was explored based on 30,995,833 ILI cases, 286,916 Flu A cases, and 177,881 Flu B cases from 554 sentinel hospitals in 30 provinces and municipalities in China in 2010–2017. This study analyzed the influence of temperature on influenza in different subtypes and regions to provide a scientific theoretical basis for preventing and controlling influenza.

## 2. Methods

**Figure 1:**
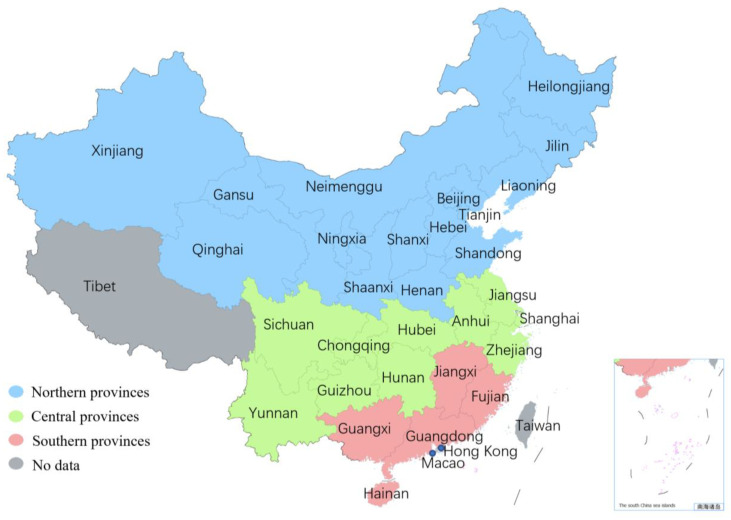
Map of China’s provincial divisions. Blue color denotes northern China, green color denotes central China, red color denotes southern China, and gray color denotes no data.

China lies in East Asia and the western coast of the Pacific Ocean. The territory is vast, with a total land area of approximately 9.6 million square kilometers. China has various climates. The eastern half has a large range of monsoon climates, and the Qinghai–Tibet Plateau is high in altitude and large in area, forming a unique alpine climate. The northwest area is located in a remote inland area and has a westerly inland arid climate. Based on the seasonal characteristics of influenza in different regions [12], the provinces and municipalities were divided into three influenza epidemiological regions: northern, central, and southern China, as shown in Figure 1. Northern China included Beijing, Gansu, Hebei, Heilongjiang, Henan, Jilin, Liaoning, Inner Mongolia, Ningxia, Qinghai, Shaanxi, Shandong, Shanxi, Tianjin, and Xinjiang. Central China included Anhui, Chongqing, Guizhou, Hubei, Hunan, Jiangsu, Shanghai, Sichuan, Yunnan, and Zhejiang. Southern China included Fujian, Guangdong, Guangxi, Hainan, and Jiangxi.

### 2.1. Sources of Influenza Data

This study used weekly influenza case reports from the National Influenza Center of China from 2010 to 2017. The dataset provided the number of visits, ILI, specimens tested, and laboratory-confirmed influenza cases from 554 sentinel hospitals in 30 provinces and municipalities of mainland China. Flu A included H1N1, H3N2, (H1N1)pdm09, and those without detectable subtypes. Flu B included Yamagata, Victoria, and those without any detectable subtypes. A standard case of ILI was defined as having a body temperature greater than 38 °C with cough or sore throat and no available alternative diagnosis [12]. The ILI data are highly correlated with the influenza data [14], so it is widely evaluated in the surveillance and reporting of influenza cases by authorities such as the WHO to obtain a complete and accurate picture of influenza activity [15,16].

Several indicators were used to demonstrate influenza activity in China [17]. First, the ILI rate was defined as the number of ILI cases divided by the number of outpatient visits multiplied by 10,000. Second, the positive rate for Flu A or Flu B was defined as the number of laboratory-confirmed influenza cases divided by the number of samples tested. Third, the incidence rate of Flu A or Flu B was defined as the rate of ILI times the positive rate of Flu A or Flu B, which more precisely characterizes influenza virus infection [18]. The weekly incidence rate was interpolated to the daily incidence rate using a natural cubic spline curve [19].

### 2.2. Sources of Climate and Population Data

Meteorological data were obtained from the National Meteorological Science Data Center (https://data.cma.cn/ (accessed on 20 August 2022)). Data from 4 January 2010 to 31 December 2017 were used, including the daily average temperature (°C), relative humidity (%), wind speed (m/s), and atmospheric pressure (hPa). Data from 839 weather stations distributed in 30 provinces and municipalities were summarized as provincial daily average data. Population data for each province were derived from the Seventh National Census Bulletin of the National Bureau of Statistics (http://www.stats.gov.cn/ (accessed on 22 August 2022)). The national legal holiday date was sourced from The General Office of the State Council (http://www.gov.cn/zhengce.htm (accessed on 22 August 2022)). For the school winter and summer vacation, because the vacation times in each province varied, the winter and summer vacation times were unified from January 15 to February 15 and July 15 to August 31 [20] for the convenience of statistics.

### 2.3. Statistical Analysis

Because temperature, relative humidity (RH), wind speed, air pressure (AP), and the number of ILI, Flu A, and Flu B cases did not follow a normal distribution, the Spearman rank correlation test was used to examine the relationship between meteorological factors and the influenza incidence rate.

A distributed lag nonlinear model (DLNM) was used to analyze and compare the exposure-lag response of the daily mean temperature on the rate of ILI and the incidence rates of Flu A and Flu B in different regions. DLNM was developed by Gasparrini and Armstrong [21]. It is widely used to evaluate relationships between meteorological factors and health effects. This model can describe the complex nonlinear and lag-dependence relationships by combining two defining conventional functions. It selects the primary functions of exposure–response and lag–response to form a cross-basis function, thereby reflecting the nonlinear model of the exposure–lag–response relationship. Because the daily incidence rate of influenza is close to the Poisson distribution, the temperature has a lag effect on the incidence of influenza according to previous studies [22]. Therefore, DLNM can better assess the potential nonlinear effects of temperature on seasonal influenza activity with delayed effects.

In this study, natural cubic splines were used to simulate exposure–response and lag–response curves. To better fit the model, quasi-Akaike information criterion (qAIC) was used to determine the number of covariates and nodes. A smaller qAIC value indicates better model fitting. Four node numbers were selected for the exposure–response curve, and two node numbers were selected and placed at equal spacing on a logarithmic scale for the lag–response curve. The Quasi-Poisson link function was used to fit the cross-product matrix of influenza incidence rate and meteorological data. To control for seasonality and long-term trends, we included a time period of two degrees of freedom per year in the model [23]. RH, wind speed, and AP were included as confounders in the model. The final statistical model is as follows:

log[E(Y_t_)] = *α* + *β*(TEM_t,l_, lag = 14) + ns(X_i_, df = 3) + ns(Time_t_, df = 2/year) + *γ*DOW_t_ + *η*Holiday_t_ (1)

where Y_t_ is the rate of ILI or the incidence rate of Flu A/Flu B on day t; α is the intercept term; TEM_t,l_ is the two-dimensional matrix about temperature obtained by the ‘cross-basis’ function; *β*, *γ,* and *η* are the coefficients of explanatory variables. ns is a natural cubic spline function; X_i_ represents the three meteorological factors RH, wind speed, and AP, and their degrees of freedom were set to 3. Time_t_ represents the long-term trend, and its degree of freedom was set to 2/year; DOW_t_ is a dummy variable for the day of the week; Holiday_t_ is the holiday effect, including national statutory holidays, winter, and summer holidays, and the holiday was set as 1, while the non-holiday was set as 0.

Based on previous research reports [23], 14 days was used as the maximum lag. The relative risk (RR) of the cumulative effect and its 95% confidence interval (CI) were calculated, and the reference value was set as the lowest risk of the fitting curve [24]. Moreover, the RR of influenza at different temperatures (low, medium, and high temperatures were 10%, 50%, and 90% quantiles of the region’s temperature, respectively), and lag times (lag = 0, 7, and 14 days) were estimated.

To assess the stability of the model, we performed a sensitivity analysis by varying the maximum lag time from 12 to 14 days. All analyses were performed using R (Ross Ihaka and Robert Gentleman, Guangdong, China), version 4.2.0.

## 3. Results

### 3.1. Results of Descriptive Analysis

We detected 30,995,833 ILI cases during the study period, of which 286,916 were Flu A, accounting for 0.93% of the total ILI cases, and 177,881 were Flu B, accounting for 0.57% of the total ILI cases (Table S1 in the Supplementary Materials). Table 1 summarizes the incidence rate of influenza and the characteristics of the climate variables in northern, central, and southern China from 2010 to 2017. The daily average temperature was 9.08 ± 11.35 °C in northern China, 16.38 ± 7.76 °C in central China, and 21.08 ± 6.36 °C in southern China. Since the data were skewed, we used the Kruskal–Wallis H test. The test results showed significant differences in meteorological factors and median influenza incidence rate among the three areas (*p* < 0.05). The median incidence rates of Flu A and Flu B were highest in southern China and lowest in northern China. The median rate of ILI was highest in southern China and slightly lower in central China than in northern China.

**Figure 2:**
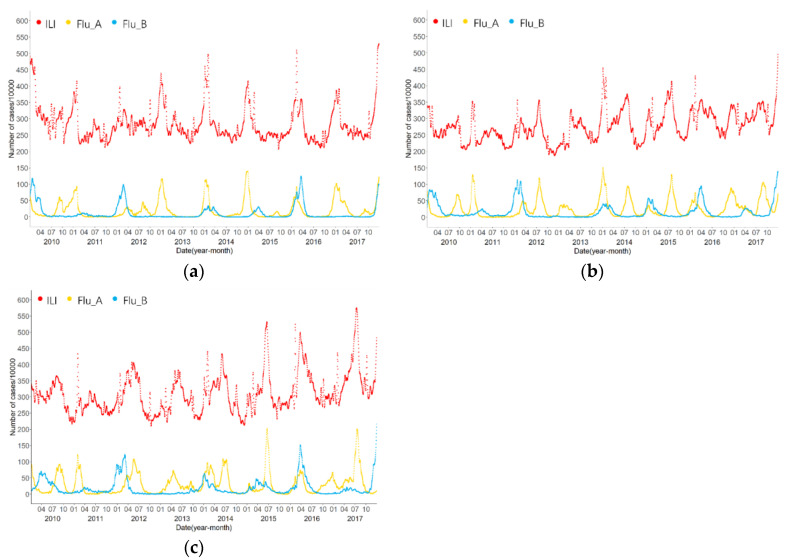
Influenza trends in the three regions of China, 2010–2017. (**a**) Northern China; (**b**) Central China; (**c**) Southern China. The red, yellow, and blue dots show the rate of ILI, the incidence rate of Flu A, and that of Flu B, respectively. Abbreviations: ILI, influenza-like illness; Flu A, influenza A; Flu B, influenza B.

Figure 2 illustrates the monthly influenza incidence rate trend from 2010 to 2017, showing a cyclical trend. ILI and Flu A peaked in winter and spring (November to March) as well as summer (July to September) in most years, respectively, while Flu B did not peak in summer, and only one peaked in winter and spring (November to May) except in the winter and spring of 2012–2013. The epidemic trends in the three regions were slightly different; central and southern China had more and higher peaks than northern China. Moreover, the ILI rate in southern China had an increasing trend each year.

Figure S1 in the Supplementary Materials showed the monthly variation trends of four meteorological factors, including temperature, RH, wind speed, and AP, in the three regions from 2010 to 2017. Temperature and AP in the three regions all had clear periodic changes. Additionally, the periodic variation trends of RH and wind speed were obvious in northern China but not in central and southern China. Figure S2 in the Supplementary Materials showed the incidence rate of ILI in each province of China from 2010 to 2017 with a heat map to compare differences between the different provinces. This was standardized to the number of ILI cases per 100,000 population according to the total population of each province. The provinces with more ILI cases were mainly concentrated in the eastern and southern coastal areas and northwest areas, and the number of ILI cases increased yearly.

### 3.2. Analysis of Correlation

**Figure 3:**
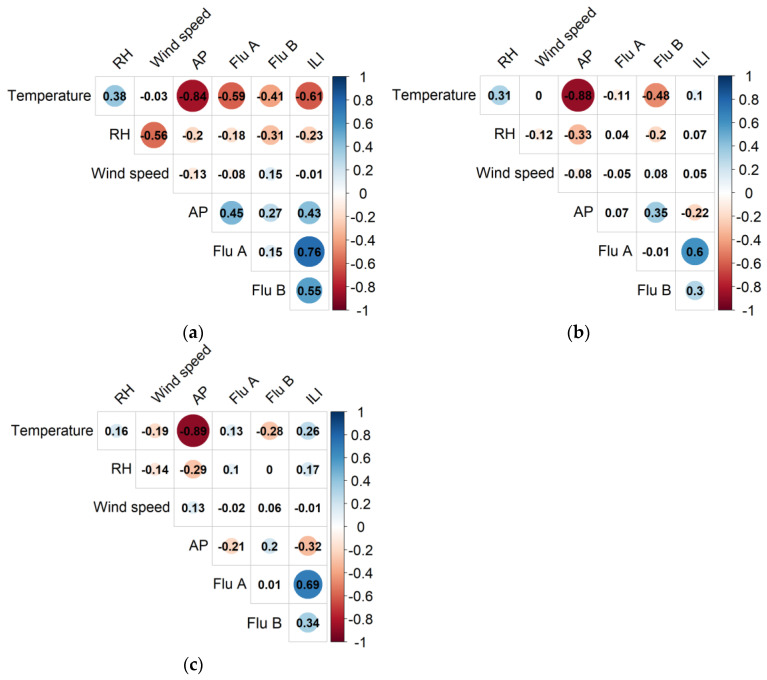
Results of Spearman’s rank correlation analysis between ILI/Flu A/Flu B and meteorological factors. (**a**) Northern China; (**b**) Central China; (**c**) Southern China. The numbers in the figure are Spearman’s rank correlation coefficients. A darker circle indicates a larger absolute value of the coefficient. Two variables with insignificant correlations are represented by blanks. Abbreviations: ILI, influenza-like illness; Flu A, influenza A; Flu B, influenza B; RH, relative humidity; AP, air pressure.

Based on the Spearman rank correlation analysis shown in Figure 3, in northern China, the incidence rates of ILI, Flu A, and Flu B significantly correlated with other meteorological factors (all *p* < 0.05), but the correlation between ILI rate and wind speed was not significant. The incidence rates of ILI, Flu A, and Flu B negatively correlated with temperature and RH but positively correlated with AP. The incidence rate of Flu A negatively correlated with wind speed, but the incidence rate of Flu B positively correlated with wind speed.

In central China, the incidence rates of ILI, Flu A, and Flu B significantly correlated with four meteorological factors (all *p* < 0.05). The incidence rate of Flu A negatively correlated with temperature and wind speed and positively correlated with RH and AP. The incidence rate of Flu B negatively correlated with temperature and RH, and positively correlated with wind speed and AP. The ILI rate positively correlated with temperature, RH, and wind speed, and negatively correlated with AP.

In southern China, the incidence rates of Flu A and ILI positively correlated with temperature and RH and negatively correlated with AP. The incidence rate of Flu B negatively correlated with temperature and positively correlated with wind speed and AP (all *p* < 0.05), but the absolute value of the correlation coefficient was small. The *p*-values for Spearman’s rank correlations are detailed in Table S2 in the Supplementary Materials.

### 3.3. Overall Effect of Temperature on ILI/Flu A/Flu B

We conducted sensitivity analyses to assess the robustness of model selection, as shown in Figure S3 in the Supplementary Materials. By varying the maximum lag time (12–14 days), the cumulative exposure–response relationship between temperature and risk of influenza did not significantly change. Therefore, the model realistically reflected the effect of temperature on the risk of influenza.

**Figure 4:**
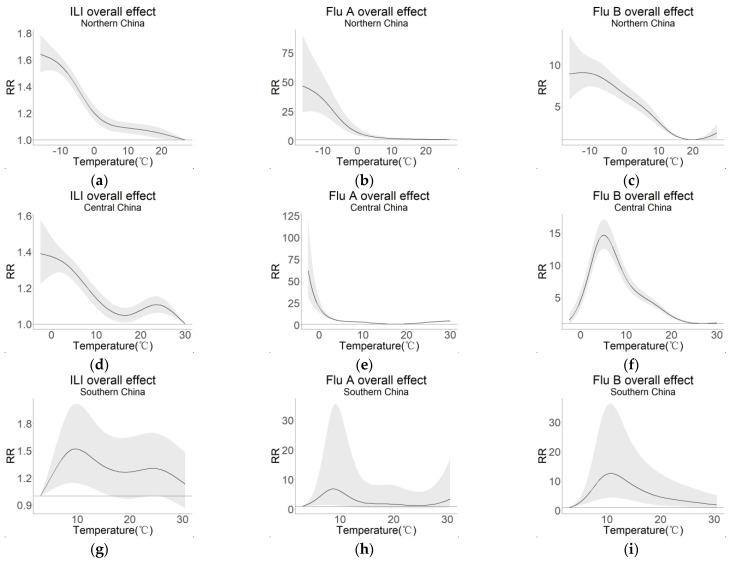
Overall effects between ILI, Flu A, and Flu B and temperature in different regions and 95% confidence intervals. (**a**) ILI overall effect in northern China; (**b**) Flu A overall effect in northern China; (**c**) Flu B overall effect in northern China; (**d**) ILI overall effect in central China; (**e**) Flu A overall effect in central China; (**f**) Flu B overall effect in central China; (**g**) ILI overall effect in southern China; (**h**) Flu A overall effect in southern China; (**i**) Flu B overall effect in southern China. The shaded area represents the 95% confidence interval. Abbreviations: ILI, influenza-like illness; Flu A, influenza A; Flu B, influenza B; RR, relative risk.

Figure 4 shows the overall effects of ILI, Flu A, and Flu B as well as temperature in the three regions. At a lag time of 14 days, with the temperature at the lowest RR as a reference, DLNM had a nonlinear relationship between temperature, ILI rate, and incidence rate of Flu A and Flu B in all three regions.

In northern China, the RR values of ILI, Flu A, and Flu B all showed a decreasing trend, with the highest RR at −16 °C of 1.640 (95% CI: 1.506–1.786), 46.735 (95% CI: 24.193–90.281), and 8.912 (95% CI: 5.837–13.608), respectively. In central China, the RR of ILI had a roughly decreasing trend but peaked at 23.5 °C. The RR of Flu A decreased rapidly at low temperatures, then decreased slowly with increasing temperature, and finally increased slowly above 18 °C. The RR of Flu B showed an inverted V-shape and reached the maximum at 5 °C with 14.697 (95% CI: 12.603–17.139). In southern China, the RR of ILI increased and then decreased slowly with temperature and peaked at 9.5 °C and 24.5 °C with 1.519 (95% CI: 1.144–2.018) and 1.305 (95% CI: 1.004–1.696), respectively. The RR of Flu A increased at low temperatures, then decreased with increasing temperature, reached a peak at 8.5 °C, and slowly increased again above 24.5 °C. The RR of Flu B showed an inverted V-shape and reached a maximum value of 12.573 (95% CI: 4.349–36.343) at 10.5 °C.

### 3.4. Relationship between Lag Time and Influenza Risk at Different Temperatures

**Figure 5:**
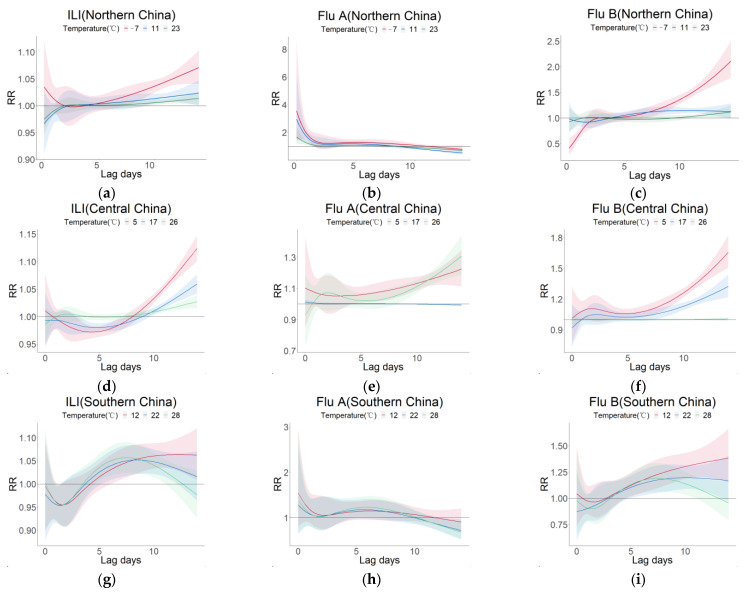
Relationship between the lag times of ILI, Flu A, and Flu B in different regions at specific temperatures (10%, 50%, 90%) with RR. The red line indicates the 10% quantile of the temperature in the area, the green line indicates the 50% quantile of the temperature in the area, and the blue line indicates the 90% quantile of the temperature in the area. (**a**) ILI in northern China; (**b**) Flu A in northern China; (**c**) Flu B in northern China; (**d**) ILI in central China; (**e**) Flu A in central China; (**f**) Flu B in central China; (**g**) ILI in southern China; (**h**) Flu A in southern China; (**i**) Flu B in southern China. The shaded area represents the 95% confidence interval. Abbreviations: ILI, influenza-like illness; Flu A, influenza A; Flu B, influenza B; RR, relative risk.

As shown in Figure 5, we investigated the relationship between lag time and influenza risk in the three regions at low (10% quantile of temperature in the region), moderate (50% quantile of temperature in the region), and high temperatures (90% quantile of temperature in the region). We found that the relationship between RR and lag days was similar at different temperatures.

In northern China, the RR of ILI and Flu B increased with lag time at different temperatures by and large, and the RR of −7 °C was greater than that of 11 °C and 23 °C. With a lag of 14 days, the RR of ILI and Flu B reached a maximum at −7 °C (Figure 5a,c). In contrast, the RR of Flu A showed a decreasing trend at different temperatures, and the maximum value was reached when the lag was 0 (Figure 5b). Additionally, there was a rapid decrease in the RR of ILI at low temperatures as well as in the RR of Flu A during the first two lag days.

In central China, the RR of ILI, Flu A, and Flu B had a decreasing trend at first and then increased with lag days at different temperatures. The RR of ILI and Flu B at 5 °C was higher than those at 17 °C and 26 °C, and the maximum values were reached with 14 lag days, respectively (Figure 5d,f). The RR of Flu A at 17 °C was lower than that at 5 °C and 26 and reached a maximum when the lag was 14 days (Figure 5e).

In southern China, the RR of ILI, Flu A, and Flu B decreased rapidly during the first two lag days, then increased, and finally decreased with the lag time at different temperatures. The RR of ILI reached a peak value of 1.063 (95%CI: 1.018–1.018) with a lag of 13.2 days at 12 °C, 1.052 (95%CI: 1.021–1.083) with a lag of 8.4 days at 22 °C, and 1.057 (95%CI: 1.024–1.090) with a lag of 7.6 days at 28°C (Figure 5g). The RR of Flu B increased with the lag time at 12 °C and was larger than those at 22 °C and 28 °C. At 22 °C and 28 °C, the RR peaked at a lag of 10 days and 7.4 days, respectively (Figure 5i).

## 4. Discussion

This study analyzed the association between temperature and influenza incidence across a wide range of regions in China over a long period from 2010 to 2017. The large data size and the long time span of our study demonstrated the good representativeness of the findings. Our results showed that temperature affected influenza activity in several ways. In northern China, low temperatures significantly increased the risk of ILI, Flu A, and Flu B. In central and southern China, both low and high temperatures increased the risk of ILI and Flu A, while only low temperatures affected Flu B activity.

### 4.1. Epidemiological Characteristics of Influenza

ILI, Flu A, and Flu B were all distinctly seasonal, but the epidemiological trends in the three regions were slightly different, with more influenza peaks and a higher number of cases in southern China. Our results showed that influenza activity in northern China typically had only one peak in winter and spring, whereas central and southern China had peaks in both winter and summer (Figure 2). This is consistent with reports from the Chinese Center for Disease Control and Prevention that the peak of influenza in the northern region generally occurs in winter and spring, that is, from December to January each year, while the southern region generally has two influenza peaks in winter and summer [25]. Moreover, Flu B has one peak each year in winter and spring in the three regions. During the winter of 2012–2013, Flu B activity was low, which might reflect the complex interactions between population immunity and viral evolution of Flu B lineages [26,27]. In contrast, Flu A has two large peaks in both winter and summer, which is consistent with previous studies showing that Flu A activity was also elevated in summer in subtropical cities in China [28].

Nationally, provinces and municipalities with more ILI reports were largely located in southeastern China (Figure S2 in Supplementary Materials). Most coastal cities in the southeast are trade and transportation hubs with high population density and mobility. Increased influenza transmission has been reported to be associated with population density [29,30]. Additionally, owing to the rapid economic development of coastal areas, influenza detection systems are better and more sensitive [12]. This may also result in more influenza cases being detected by sentinel hospitals in these provinces.

### 4.2. The Overall Effect of Temperature on Influenza

Temperature can influence various aspects of influenza virus transmission, but the mechanism is complex [31]. The overall effect estimated in our study suggested that in northern China, low temperatures increased the risk of influenza (Figure 4a–c), which was consistent with studies in high latitudes. A study in Finland found that between −22.8 and 22.0 °C, each 1 °C drop in temperature increased the risk of influenza virus infection by 11%, with 74% of influenza virus infections occurring in the −10 to 5 °C temperature range [32].

We also found that high temperatures increased the risk of influenza in central and southern China (Figure 4d,g), which is similar to the results of a study in Hong Kong [6]. They found that at high temperatures, for every 2 °C rise in temperature from May to November, the number of days favorable for Flu A transmission increased from 58% to 71%.

Moreover, the RR of Flu B peaked at moderately low temperatures in both central and southern China but not in northern China. This trend was consistent with the finding in a southern city in China, Shenzhen, [33] that the risk of Flu B was highest under moderately cold conditions rather than very low temperatures.

In contrast to our findings, a similar study by Zhang et al. [4] of 30 cities in China found an N-shaped relationship between temperature and cumulative relative risk of influenza in northern China, with two peaks of influenza risk at both moderately low (around 5 °C) and high temperatures (around 30 °C). This result was not consistent with the epidemiological characteristics of influenza in northern China where influenza generally has only one peak in winter and spring (around −10 °C) shown in Figure 2a. In our results, as shown in Figure 4a, the RR of ILI in northern China had the highest value at -16 °C, which led to only one peak of the incidence rates of influenza during the winter. Moreover, they found an inverted V-shaped relationship between temperature and cumulative relative risk of influenza in southern China, with a peak at approximately 10 °C. This result was also not consistent with the epidemiological characteristics of influenza in southern China where influenza has two peaks in winter and spring (around 10 °C) and summer (around 25 °C) shown in Figure 2c. In our study, as shown in Figure 4g, the RR of ILI in southern China had peaks at both low (9.5 °C) and high (24.5 °C) temperatures, which resulted in two peaks of influenza incidence rates in summer and winter. The differences between the findings of our study and those of Zhang et al. may be due to the discrepancy in the data sources [34] and computation periods. The database of our study was 30,995,833 ILI cases, 286,916 Flu A cases, and 177,881 Flu B cases from 554 sentinel hospitals in both urban or rural areas in 30 provinces and municipalities in 2010–2017, while that of Zhang et al. was 1,712,970 cases from 30 cities in 2016–2019.

#### 4.2.1. Mechanisms of Low and High Temperatures Affecting the Onset of Influenza

Studies have shown that cold temperatures contribute to the spread of influenza in several ways, such as by affecting virus survival and transmission, influencing host susceptibility, and altering human behavior and the environment [9,35]. Various research hypotheses explain the relationship between low temperatures and influenza. First, when the temperature decreases, the stability of influenza virus particles increases, and the physical properties of the viral envelope change, thus promoting the survival and spread of influenza [36]. Second, when people inhale cold air, the nasal epithelium is cooled, which can weaken the ability of the respiratory tract to defend against infection, such as mucociliary clearance and phagocytic activity of leukocytes [22]. Additionally, when it is cold outside, people are more inclined to stay indoors and not go outside, thereby increasing the probability of human contact [37,38,39]. Cooler outdoor temperatures may also lead to an increase in the use of indoor heating equipment, reducing indoor humidity and promoting the spread of influenza [40]. Some animal experiments have shown that leukocytes and IgM are reduced in cold environments, thereby suppressing the immune function of experimental animals [8]. However, the innate immunity of guinea pigs housed at 5 °C is not greatly compromised compared to those housed at 20 °C [7].

The risk of influenza also increased in hot environments in the central and southern regions. Due to extreme outdoor heat, people go outside less often and use indoor air conditioning more frequently, which promotes person-to-person contact and the spread of influenza [41]. Tamerius et al. [42] found that epidemics in tropical and subtropical regions occurred during periods of high temperature and humidity. In addition, Grigorieva et al. [43] found that the combined effect of high temperature and air pollution promotes the occurrence of respiratory diseases such as influenza. From an experimental point of view, Moriyama et al. [44] also demonstrated that the exposure of mice to high ambient temperatures reduced their food intake and weakened their adaptive immune response to influenza virus infection.

#### 4.2.2. Differences between Flu A and Flu B

The RR of Flu A tended to increase at high temperatures in central and southern China, whereas that of Flu B did not (Figure 4). A study in Hong Kong [6] also found that hot and humid conditions were associated with a higher activity of Flu A but were associated with only a moderate increase in the activity of Flu B. Moreover, we observed an increasing trend in the magnitude of summer peaks with Flu A rather than Flu B. The variability of Flu A and Flu B may be related to differences in the characteristics of the two viruses, such as surface glycoproteins, which may account for their different responses to environmental factors. As Woese [45] suggested, the thermal denaturation of proteins and nucleic acids was the mechanism of virus inactivation, and environmental factors might lead to changes in the surface glycoproteins of the virus, thus affecting its stability [46].

### 4.3. Relationship between Lag Time and the Risk of Influenza

We found that the RR of influenza in all three regions had a significant downward trend for the first two lag days and then slowly increased, reaching a maximum at a lag of 14 days (Figure 4), which is consistent with the findings of a study in a mid-eastern province in China, Jiangsu [23]. They thought that the peak in RR for influenza at lag 0 days was because people with underlying disease or immunodeficiency were more susceptible to influenza virus infection, and after these people recovered, others gradually became infected after the incubation period, thus causing a slow rise in subsequent RR. Moreover, the RR of ILI, Flu A, and Flu B generally increased with the lag time. A study in China [47] reported that the incubation period before the onset of influenza was up to 13 days. The incubation period may explain, to some extent, why RR was higher on a longer lag day in our study.

### 4.4. Limitations

Our study has some limitations. First, other factors, such as economic level, industrial level, agricultural level and medical level, were not considered in this study [48,49] due to the lack of data for the 554 sentinel hospitals in 30 provinces and municipalities. Second, the data on influenza incidence were derived from case reports from sentinel hospitals, which may not fully reflect the number of influenza cases; hence, the accuracy of the data may be affected. Third, we did not consider the effects of host susceptibility and seasonal influenza vaccine application on influenza incidence. Finally, as a national study, our findings may be subject to ecological fallacies; therefore, more precise data or experimental studies are needed for future exploration.

## 5. Conclusions

In conclusion, our study found that low temperatures significantly increased the risk of ILI, Flu A, and Flu B in northern China, while in central and southern China, both low and high temperatures increased the risk of ILI and Flu A, while only low temperatures increased the risk of Flu B. Moreover, the temperature had a lag effect on influenza, and the longer the lag time, the greater the risk. Therefore, it is recommended that temperature be integrated into the Influenza Early Warning and Forecasting System to improve the accuracy of influenza warnings. Local governments or CDCs can advise the public on self-prevention and control of influenza according to the temperature characteristics of influenza onset.

## Figures and Tables

**Table 1:** Descriptive summary of meteorological variables and influenza rates in different regions.

		Mean Temperature ^a^ (°C)	Mean Relative Humidity ^a^ (%)	Mean Wind Speed ^a^ (m/s)	Mean Atmosphere Pressure ^a^ (hPa)	Incidence Rate of Flu A	Incidence Rate of Flu B	ILI Rate
Northern China	Mean ± sd	9.08 ± 11.35	58.91 ± 10.27	2.30 ± 0.46	929.54 ± 6.64	19.96 ± 28.16	10.86 ± 22.22	286.00 ± 54.40
	5%	−8.81	41.74	1.68	919.29	0.20	0.02	228.62
	25%	−1.64	51.02	1.96	923.98	1.45	0.42	250.40
	50%	10.80	59.39	2.23	929.60	7.48	1.53	270.14
	75%	19.78	67.55	2.58	934.74	24.71	7.93	303.63
	95%	23.95	74.30	3.15	940.30	88.53	64.45	401.23
Central China	Mean ± sd	16.38 ± 7.76	73.90 ± 7.43	1.95 ± 0.41	955.13 ± 7.00	25.95 ± 28.76	15.81 ± 23.22	278.4 ± 46.95
	5%	4.01	59.94	1.38	944.62	0.87	0.71	211.20
	25%	9.51	69.22	1.66	949.10	4.24	2.25	244.08
	50%	17.23	74.65	1.90	955.28	15.08	5.54	273.20
	75%	23.22	79.46	2.18	960.63	36.31	19.40	307.73
	95%	27.60	84.57	2.71	966.55	90.46	74.63	357.51
Southern China	Mean ± sd	21.08 ± 6.36	78.61 ± 7.11	2.00 ± 0.41	993.52 ± 6.71	29.93 ± 34.04	18.40 ± 26.03	312.3 ± 61.48
	5%	9.77	65.20	1.49	983.52	0.89	0.52	234.07
	25%	15.80	74.57	1.70	987.99	4.73	3.23	270.00
	50%	22.44	79.84	1.92	993.16	17.26	8.12	301.27
	75%	26.96	83.52	2.22	998.86	44.48	21.00	344.50
	95%	28.90	88.35	2.74	100.457	94.98	77.04	429.91
*P* value ^b^	<0.001	<0.001	<0.001	<0.001	<0.001	<0.001	<0.001	<0.001

^a^: The variables were the daily average of the mean variables in the corresponding region. ^b^: *P* values were obtained by Kruskal–Wallis H test for the medians of variables in the °C three regions.

## Data Availability

Restrictions apply to the availability of these data. Data were obtained from the Chinese Center for Disease Control and Prevention and are available from the authors with the permission of Chinese Center for Disease Control and Prevention.

## Supplementary Materials

The following supporting information can be downloaded at: https://www.mdpi.com/article/10.3390/v15030594/s1, Figure S1: Meteorological trends in three regions, 2010–2017; Figure S2: Heat map of the number of ILI per 100,000 people by province in China, 2010–2017; Figure S3: Sensitivity analysis when altering the maximum lag time for 12, 13 and 14 days in the model; Figure S4: 3D plots of the relationship between ILI, Flu A, and Flu B and temperature in different regions; Figure S5: Relationship between the temperature of ILI, Flu A, and Flu B in different regions at specific lag times with RR; Table S1: Summary of the total number of cases of ILI, Flu A, and Flu B in northern, central, and southern China; Table S2: *p* value of Spearman’s rank correlation analysis between the rate of ILI/Flu A/Flu B and meteorological factors.
