# Supplementary material for: Association between Temperature and Influenza Activity across Different Regions of China during 2010–2017
Source: Viruses. 2023 Feb 21;15(3):594. doi: 10.3390/v15030594 (PMC10054176; doi:10.3390/v15030594)
Supplement: Supplementary file 1 [file viruses-15-00594-s001.zip › viruses-2214483-supplementary.pdf]

Association between temperature and influenza activity across different regions of China during 2010-2017

Table S1. Summary of the total number of cases of ILI, Flu A, and Flu B in northern, central, and southern China

	ILI	Flu A	Flu B
Total	30995833	286916	177881
Northern China	11271495	100524	60163
Central China	11676863	122585	76559
Southern China	7454224	59586	38920

Table S2. P-value of Spearman's rank correlation analysis between the rate of ILI/Flu A/Flu B and meteorological factors

	Temperature	RH	Wind speed	PRS	Flu A	Flu B	ILI
Northern China							
Temperature							
RH	0.0000						
Wind speed	0.1435	0.0000					
AP	0.0000	0.0000	0.0000				
Flu A	0.0000	0.0000	0.0000	0.0000			
Flu B	0.0000	0.0000	0.0000	0.0000	0.0000		
ILI	0.0000	0.0000	0.6163	0.0000	0.0000	0.0000	
Central China							
Temperature							
RH	0.0000						
Wind speed	0.8095	0.0000					
AP	0.0000	0.0000	0.0000				
Flu A	0.0000	0.0423	0.0087	0.0000			
Flu B	0.0000	0.0000	0.0000	0.0000	0.6865		
ILI	0.0000	0.0004	0.0033	0.0000	0.0000	0.0000	
Southern China							
Temperature							
RH	0.0000						
Wind speed	0.0000	0.0000					
AP	0.0000	0.0000	0.0000				
Flu A	0.0000	0.0000	0.2126	0.0000			
Flu B	0.0000	0.8940	0.0009	0.0000	0.5217		
ILI	0.0000	0.0000	0.6383	0.0000	0.0000	0.0000	

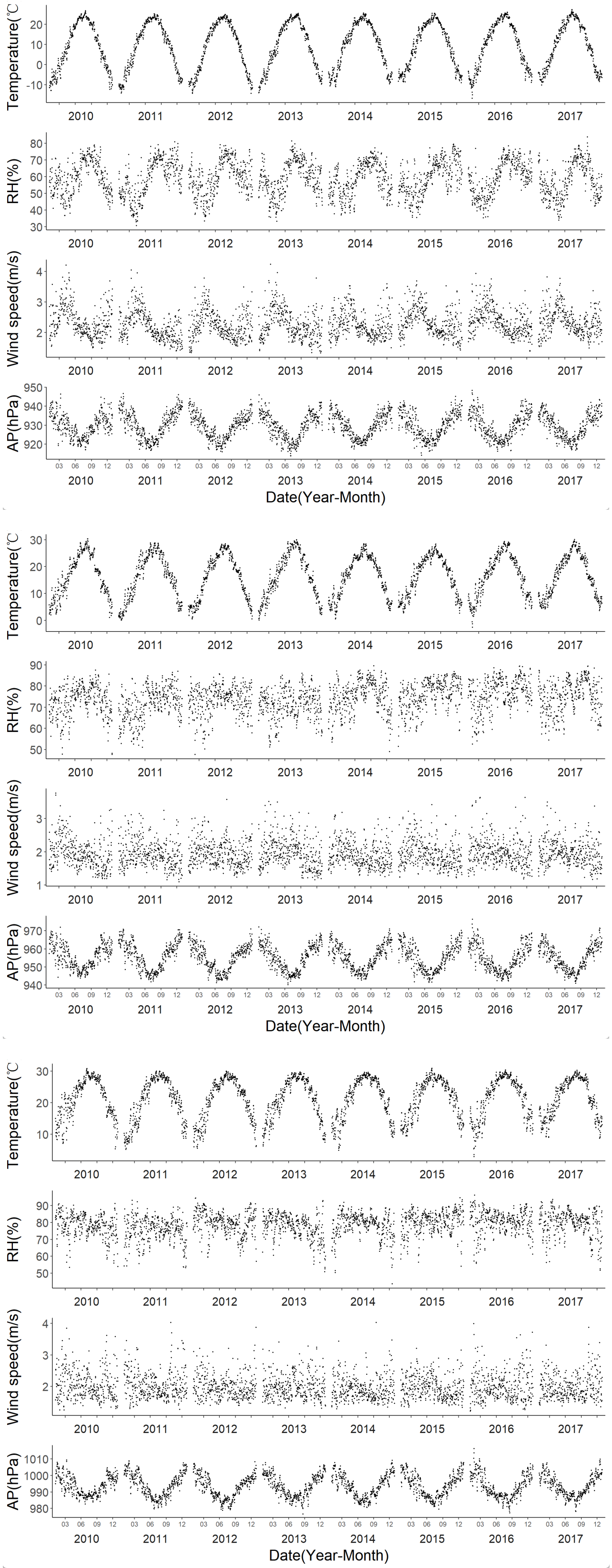

(**a**) Northern China

(**b**) Central China

(**c**) Southern China

Figure S1. Meteorological trends in three regions, 2010–2017. (**a**): Northern China; (**b**): Central China; (**c**): Southern China. Abbreviations: RH, relative risk; AP, air pressure.

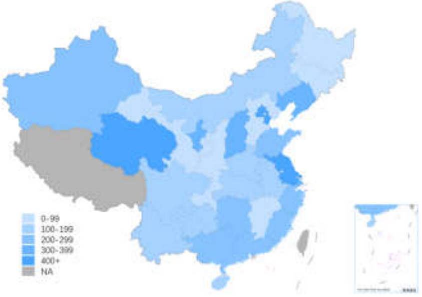

(**a**) 2010

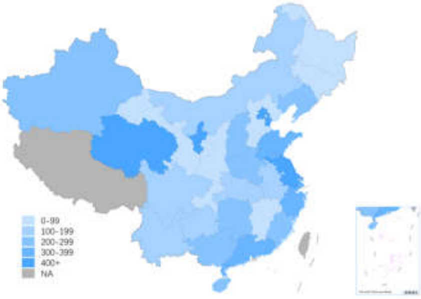

(**b**) 2011

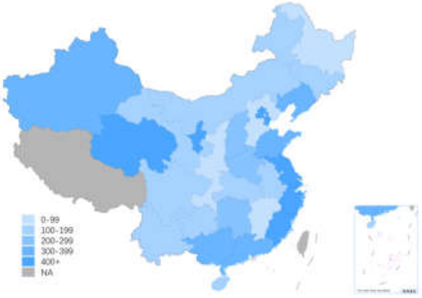

(**c**) 2012

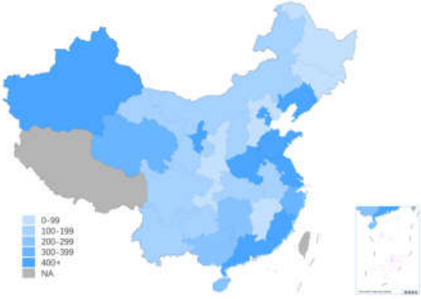

(**d**) 2013

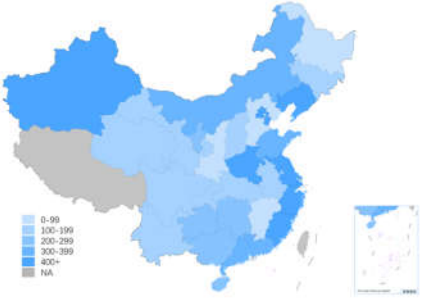

(**e**) 2014

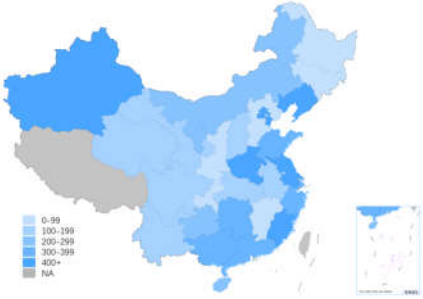

(**f**) 2015

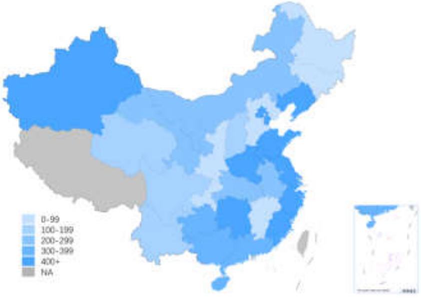

(**g**) 2016

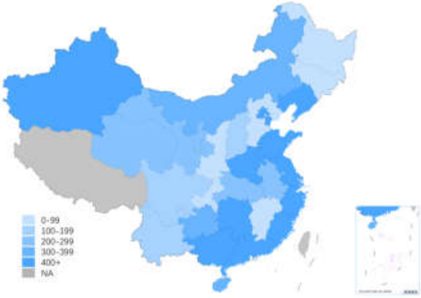

(**h**) 2017

Figure S2. Heat map of the number of ILI per 100,000 people by province in China, 2010-2017 ((**a**)-(**h**) for 2010-2017, respectively).

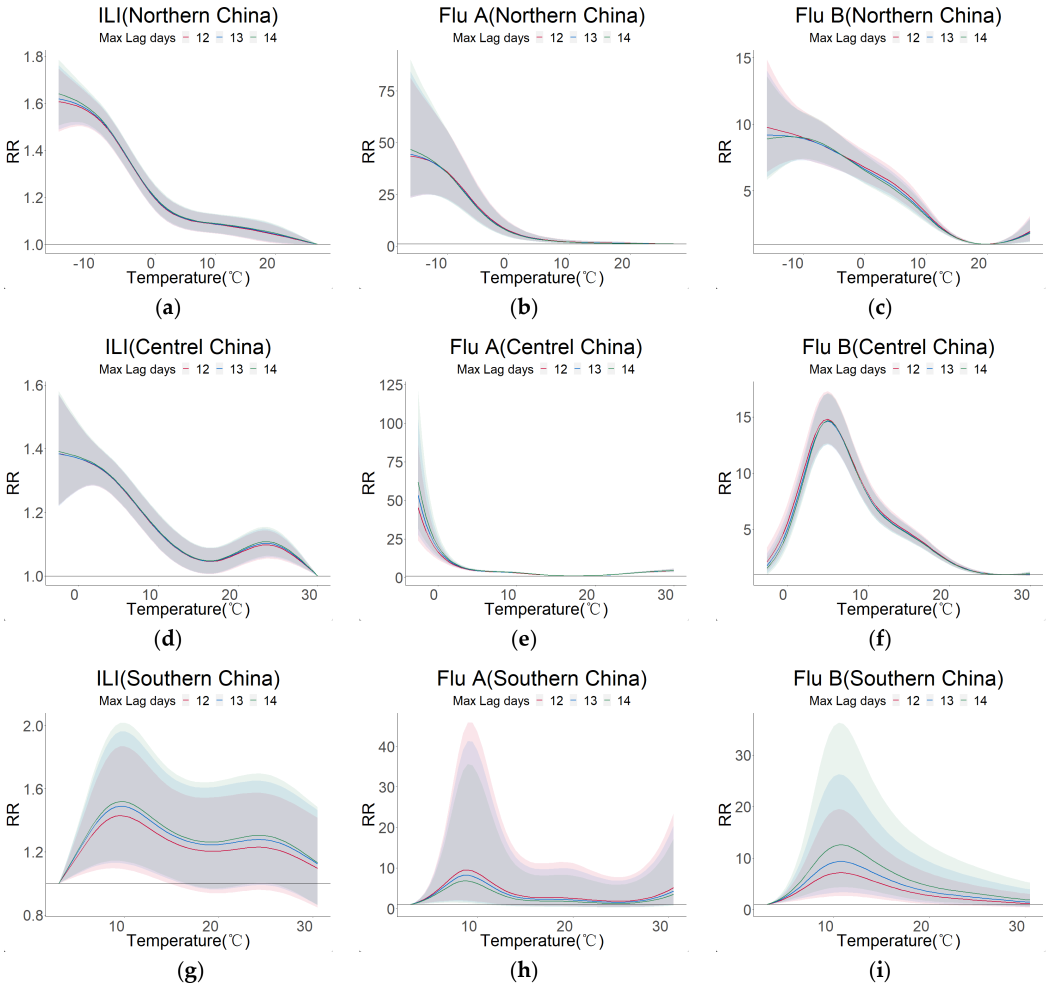

Figure S3. Sensitivity analysis when altering the maximum lag time for 12, 13, and 14 days in the model. The red color donates the maximum lag is 12; The blue color donates the maximum lag is 13; The green color donates the maximum lag is 14. (**a**): ILI in northern China; (**b**): Flu A in northern China; (**c**): Flu B in northern China; (**d**): ILI in central China; (**e**): Flu A in central China; (**f**): Flu B in central China; (**g**): ILI in southern China; (**h**): Flu A in southern China; (**i**): Flu B in southern China. The shaded area represents the 95% confidence interval. Abbreviations: ILI, influenza-like illness; Flu A, influenza A; Flu B, influenza B; RR, relative risk.

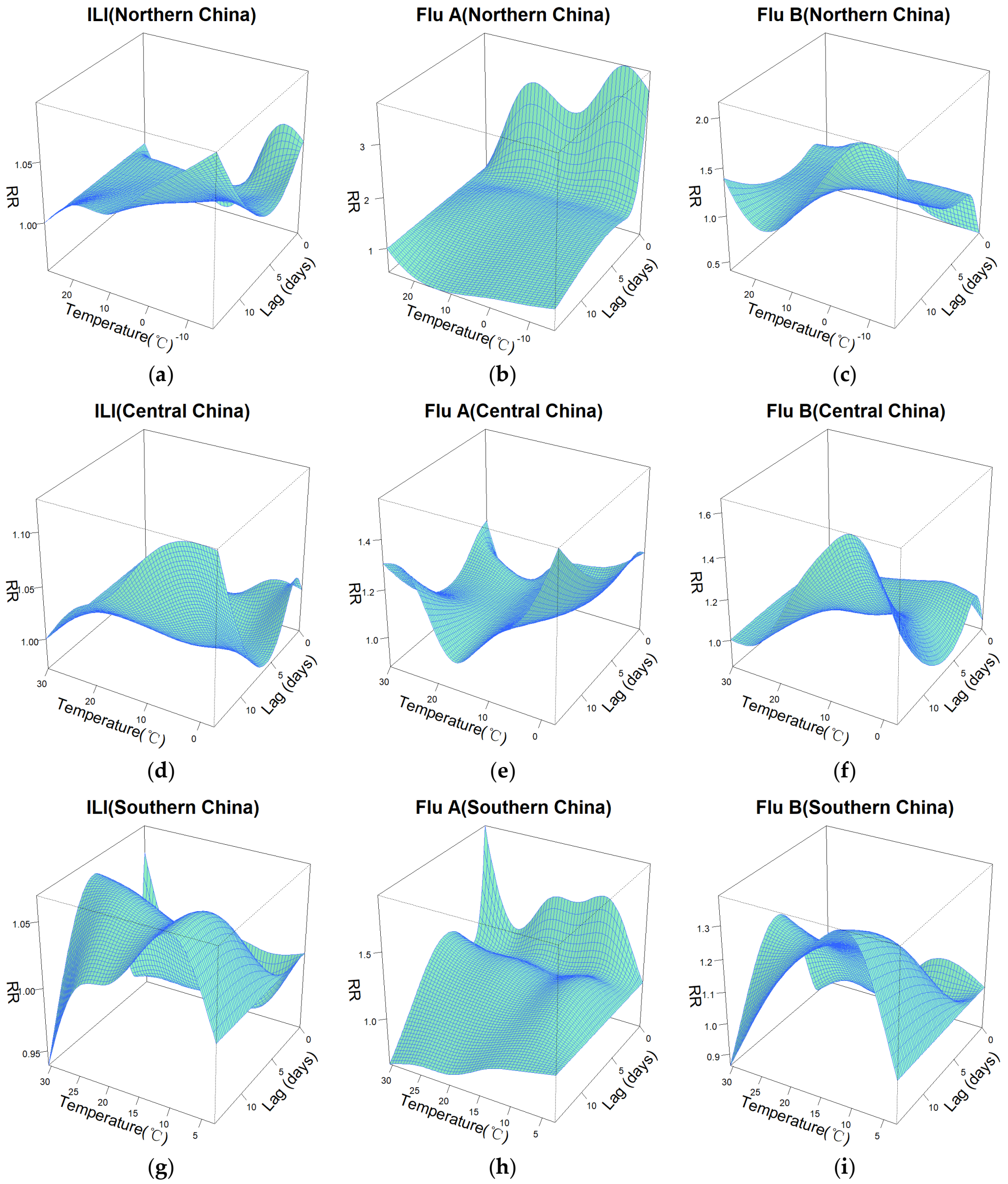

Figure S4. 3D plots of the relationship between ILI, Flu A, and Flu B and temperature in different regions. (**a**): ILI in northern China; (**b**): Flu A in northern China; (**c**): Flu B in northern China; (**d**): ILI in central China; (**e**): Flu A in central China; (**f**): Flu B in central China; (**g**): ILI in southern China; (**h**): Flu A in southern China; (**i**): Flu B in southern China. Abbreviations: ILI, influenza-like illness; Flu A, influenza A; Flu B, influenza B; RR, relative risk.

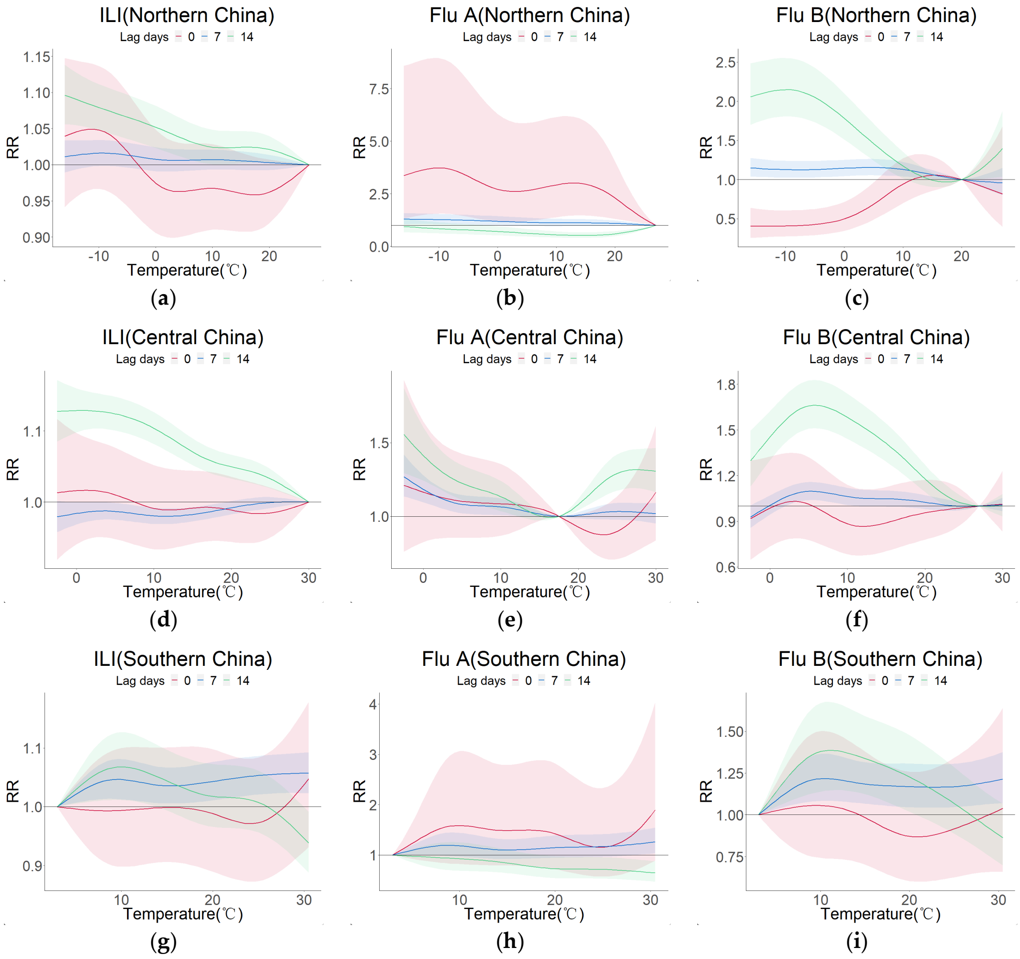

Figure S5. Relationship between the temperature of ILI, Flu A, and Flu B in different regions at specific lag times with RR. (**a**): ILI in northern China; (**b**): Flu A in northern China; (**c**): Flu B in northern China; (**d**): ILI in central China; (**e**): Flu A in central China; (**f**): Flu B in central China; (**g**): ILI in southern China; (**h**): Flu A in southern China; (**i**): Flu B in southern China. The shaded area represents the 95% confidence interval. Abbreviations: ILI, influenza-like illness; Flu A, influenza A; Flu B, influenza B; RR, relative risk.
